# The Marine Natural Compound Dragmacidin D Selectively Induces Apoptosis in Triple-Negative Breast Cancer Spheroids

**DOI:** 10.3390/md21120642

**Published:** 2023-12-15

**Authors:** Esther A. Guzmán, Tara A. Peterson, Amy E. Wright

**Affiliations:** Marine Biomedical and Biotechnology Research, Harbor Branch Oceanographic Institute, Florida Atlantic University, 5600 US 1 North, Fort Pierce, FL 34946, USA; tpitts3@fau.edu (T.A.P.); awrigh33@fau.edu (A.E.W.)

**Keywords:** marine natural products, triple-negative breast cancer, apoptosis, spheroids, reverse-phase protein arrays, invasion

## Abstract

Cancer cells grown in 3D spheroid cultures are considered more predictive for clinical efficacy. The marine natural product dragmacidin D induces apoptosis in MDA-MB-231 and MDA-MB-468 triple-negative breast cancer (TNBC) spheroids within 24 h of treatment while showing no cytotoxicity against the same cells grown in monolayers and treated for 72 h. The IC_50_ for cytotoxicity based on caspase 3/7 cleavage in the spheroid assay was 8 ± 1 µM in MDA-MB-231 cells and 16 ± 0.6 µM in MDA-MB-468 cells at 24 h. No cytotoxicity was seen at all in 2D, even at the highest concentration tested. Thus, the IC_50_ for cytotoxicity in the MTT assay (2D) in these cells was found to be >75 µM at 72 h. Dragmacidin D exhibited synergy when used in conjunction with paclitaxel, a current treatment for TNBC. Studies into the signaling changes using a reverse-phase protein array showed that treatment with dragmacidin D caused significant decreases in histones. Differential protein expression was used to hypothesize that its potential mechanism of action involves acting as a protein synthesis inhibitor or a ribonucleotide reductase inhibitor. Further testing is necessary to validate this hypothesis. Dragmacidin D also caused a slight decrease in an invasion assay in the MDA-MB-231 cells, although this failed to be statistically significant. Dragmacidin D shows intriguing selectivity for spheroids and has the potential to be a treatment option for triple-negative breast cancer, which merits further research into understanding this activity.

## 1. Introduction

Dragmacidin D is a bisindole alkaloid that was originally isolated from a deep-water marine sponge of the genus *Spongosorites* [1]. The total synthesis of this natural product has been completed [2]. This compound shows antimicrobial activity against *Escherichia coli*, with a minimum inhibitory concentration (MIC) of 15.6 µg/mL; *Bacillus subtilis*, MIC 3.1 µg/mL; *Pseudomonas aeruginosa*, MIC 62.5 µg/mL; *Candida albicans*, MIC 15.6 µg/mL; and *Cryptococcus neoformans*, MIC 3.9 µg/mL [1] (29, 6, 117, 29, and 7 µM, respectively). Dragmacidin D also has antiviral activity as it inhibits the replication of feline leukemia virus (FeLV) in vitro, with a minimum inhibitory concentration of 6.25 ug/mL (11.7 µM), as measured using an ELISA [1]. 

Dragmacidin D induces cytotoxicity in the P388 murine leukemia cell line, with an IC_50_ of 1.4 μg/mL (2.6 µM), and in the A549 human lung adenocarcinoma cell line with an IC_50_ of 4.4 μg/mL (8.3 µM), both at 72 h of treatment [1]. It was reported to be an inhibitor of serine–threonine protein phosphatases (PP), with selectivity for inhibiting PP1 over PP2A, but no details about the assay used or the concentrations tested were given in the publication [3]. A follow-up publication lists the potency of dragmacidin D as a PP inhibitor as being quite modest, in the high micromolar-to-millimolar range [4]. It was shown to inhibit the neural nitric oxide synthase (bNOS), with an IC_50_ of approximately 20 µM [5]. Dragmacidin was also tested for its ability to block resiniferatoxin-induced inflammation, inducing an 89.6% reduction in inflammation at a dose of 50 µg/ear in the mouse ear edema model of inflammation [6]. To our knowledge, dragmacidin D has not been shown to have any activity against breast cancer cells in previous studies. 

Triple-negative breast cancer (TNBC) is a collective term used to describe breast cancers that do not express the estrogen receptor (ER) or the progesterone receptor (PR) and either do not express or do not overexpress the human epidermal growth factor receptor type 2 (HER 2). Breast cancers that express any of those receptors have benefitted from targeted therapies that use those receptors as targets. These targeted therapies, along with improvements in early detection, have considerably reduced the mortality of breast cancer. TNBCs do not respond to current targeted therapies. About 10–20% of breast cancer diagnoses in the US are TNBC [7]. TNBCs are very aggressive, with their cells bearing little resemblance to normal cells, and they tend to metastasize to the brain and the lungs [7,8]. They are also associated with *BRCA1* mutations, affect premenopausal women, and are more likely to recur than other breast cancers [7,8]. TNBC is more prevalent in black women than in white women [7]. Mexican women also show a high incidence of triple-negative breast cancer [9]. There is no preferred standard form of chemotherapy for TNBC, although paclitaxel has shown benefit [8]. Even with the great improvements in reducing lethality and current effective targeted therapies, breast cancer remains the second leading cause of cancer death among women in the United States [10]. This highlights that there is still a need for new treatments to treat this disease, especially TNBC.

Notably, 3D cultures, or spheroids, are an established model that is finding resurgence. Monocultures in 3D show enhanced cell-to-cell interactions as well as cell-to-extracellular matrix interactions [11]. Furthermore, 3D cell cultures develop chemical gradients where oxygen, nutrients, and catabolites (to name a few) are present at different levels in different parts of the spheroid [11]. Hypoxia appears to be present at the center of spheroids, which is a common finding in tumors [12]. Spheroids contain different phenotypes, with quiescent, necrotic, proliferating, and hypoxic cells present [12]. More important for drug discovery, differences in the activation of signaling pathways are seen when cells are grown in 3D and compared to the same cells grown in 2D [13,14]. Very different responses are seen when evaluating potential chemotherapeutics using a 2D vs. a 3D cell culture, with the 3D model having enhanced predictability of clinical effectiveness [11,12,14]. A recent study using thirteen different TNBC cell lines showed that the drugs docetaxel, cisplatin, and epirubicin have significantly higher IC_50_s against cells grown as spheroids than against the same cells grown in 2D cultures, since growing the cells as spheroids increases the resistance to these drugs [15].

A high-content imaging multiparametric assay to measure cytotoxicity in triple-negative breast cancer cells grown as spheroids was set up to identify marine natural products from the Harbor Branch Oceanographic Institute Library that may be useful against these cancers. This assay measures the cleavage of caspase 3/7 to determine if caspase-mediated apoptosis is occurring. The cleavage of caspase 3 leads to its activation and is essential to the fragmentation of DNA, the formation of apoptotic bodies (both of which are considered hallmarks of apoptosis), and the cleavage of poly(ADP-ribose) polymerase (PARP) [16]. The assay also uses the nonpermeable nuclear dye 7 amino actinomycin D (7AAD) to follow cells that are experiencing the loss of membrane integrity, which is a marker for late apoptotic or necrotic cells [17]. Finally, by plating equal numbers of cells per well and labeling the nuclei of all cells using Hoechst 33342, the loss of cell number can be tracked. 

This assay was used to identify the ability of the compound furospinosulin 1 to induce cytotoxicity in TNBC cells grown in 3D culture [13]. To focus more on detecting compounds that induce apoptosis, the incubation time of the assay was reduced to 24 h (24 h). 

Because of the higher clinical relevance of screening in 3D cell culture (spheroids), and with a focus on finding compounds that would not have been identified in traditional screening campaigns using traditional (2D) cell culture, a screening campaign was established that focused on identifying compounds that rapidly induce apoptosis in triple-negative breast cancer cells when grown as spheroids but do not induce cytotoxicity in the same cells when grown in the more traditional 2D monocultures with a 72 h treatment. The expectation is that the compounds identified through this screening campaign may work through fundamentally different mechanisms than existing chemotherapeutic agents, thus providing new therapeutic options. We report herein the ability of dragmacidin D to induce apoptosis in MDA-MB-231 and MDA-MB-468 TNBC spheroids within 24 h of treatment while showing no cytotoxicity against the same cell lines grown in monolayers with a 72 h treatment.

## 2. Results

**Figure 1:**
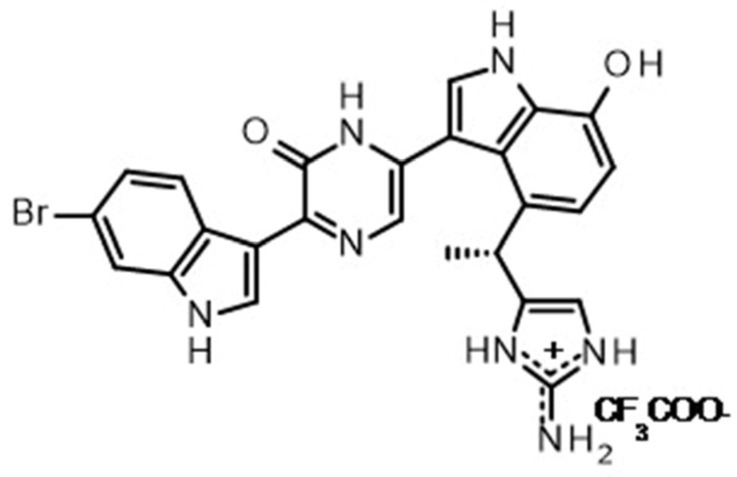
Structure of dragmacidin D.

Dragmacidin D, whose structure is shown in Figure 1, was identified as a hit in our spheroid screening assay. 

**Figure 2:**
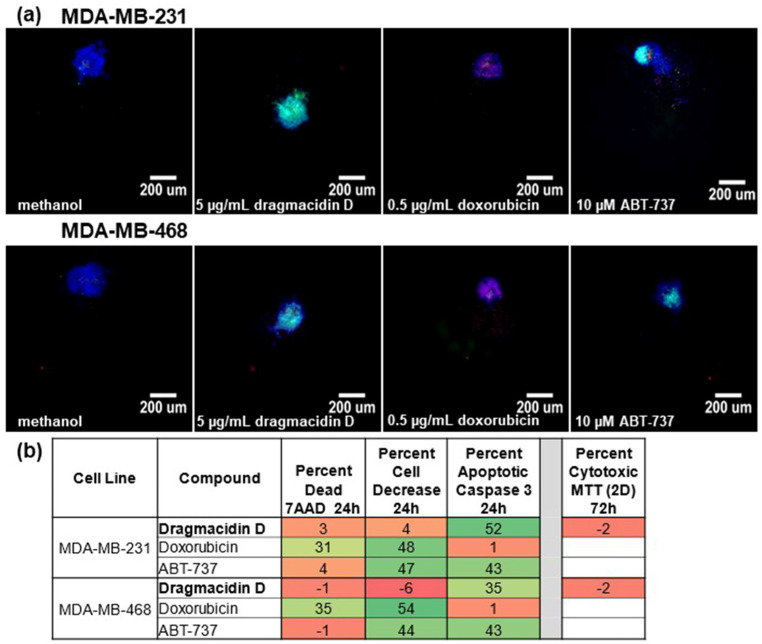
Dragmacidin D induces apoptosis in a spheroid multiparametric viability assay. Cells were plated and allowed to form spheroids overnight. Spheroids were treated with 5 µg/mL marine natural compounds or controls for 24 h. The spheroids were stained for 3 h followed by fixation. Cells were imaged in a high-content imager, and the data were analyzed using multiwavelength cell scoring. Compounds that induced caspase cleavage (green) by 30% or more, those that caused death as measured by allowing 7-amino-actinomycin D (red) to enter the cell by 30% or more, or those whose cell number decreased by 50% or more as quantitated via Hoechst 33342 (blue) staining were considered hits. Hits were confirmed by repeating the screening. Priority was given to those compounds that had caspase 3 cleavage in the spheroid assay and <20% cytotoxicity in the MTT (2D) assay: (**a**) Representative images from one screening assay are shown. Images were taken at 10× magnification. Scale bar is 200 µm. (**b**) Representative scoring values from one screening assay are presented.

This assay was performed as described previously [13], except for reducing the time of incubation with treatment from 48 to 24 h to capture more cells undergoing apoptosis. MDA-MB-231 and MDA-MB-468 triple-negative breast cancer cells were plated on special nonadherent plates and allowed to form spheroids overnight. Spheroids were treated with 5 µg/mL marine samples or controls, including solvent controls, for 24 h. At the end of the treatment, cells were stained and fixed, and images were captured and analyzed in a high-content imager. Those samples that reduced cell number by 50% or more, those that increased the number of dying or dead cells (7AAD+) by 30% or more, or those that increased the number of cells with cleaved caspase 3/7 (apoptotic cells) by 30% or more were considered hits. As a counter stain for prioritization, marine compounds were also assayed at the same concentration in a traditional MTT assay (2D). Those exhibiting caspase 3/7 cleavage in the spheroid assay with <20% cytotoxicity in 2D were given priority, as they represented compounds not likely to be identified using traditional assays. Dragmacidin D showed activity against both MDA-MB-231 and the MDA-MB-468 spheroid screening assay (Figure 2a) by increasing the cleavage of caspase 3 by more than 30% (Figure 2b). No cytotoxicity against either cell line in the standard 2D cytotoxicity assay was observed (Figure 2b). 

**Figure 3:**
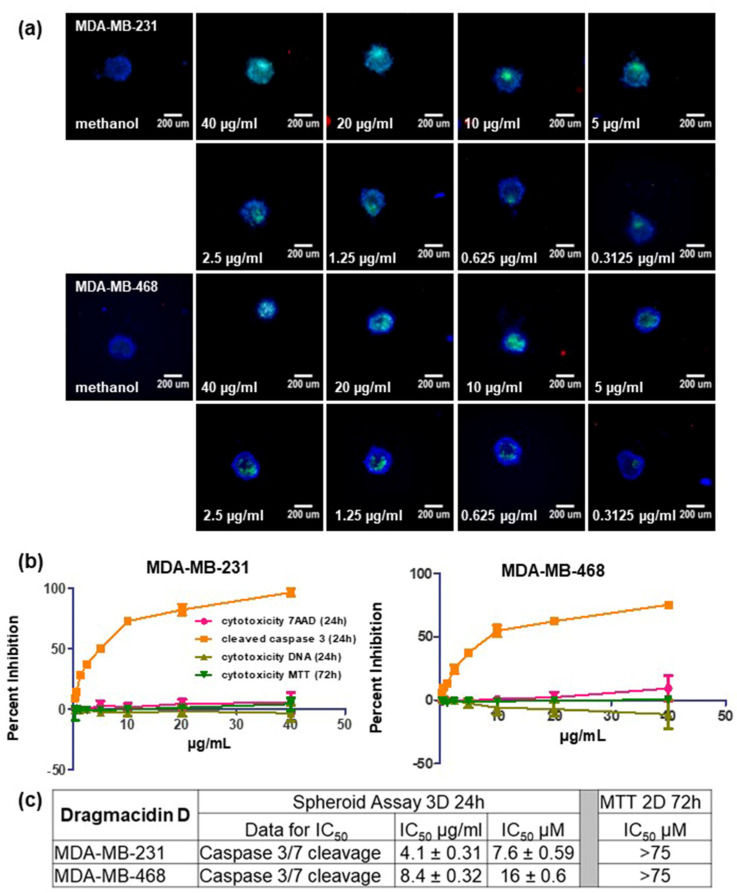
IC_50_ determination in the MDA-MB-231 and MDA-MB-468 cells. The concentration required to see 50% induction of apoptosis (IC_50_) in both cell lines was determined using serial dilutions of dragmacidin D ranging from 40 to 0.3125 µg/mL in the screening assay: (**a**) Representative images from one experiment are shown. Images were taken at 10× magnification. Scale bar is 200 µm. (**b**) The graphs show the average of three experiments ± standard deviation. The resulting percentages were normalized to solvent control and subjected to nonlinear regression. (**c**) The IC_50_ values shown represent the average of 3 experiments ± standard deviation for the spheroid assay. No cytotoxicity was seen at all in the MTT 2D assay even at the highest concentration tested (40 µg/mL ≈ 75 µM). Thus, the IC_50_ is reported as >75 µM.

Dragmacidin D was tested in the multiparametric spheroid screening assay using serial dilutions from 40 to 0.15625 µg/mL with 24 h incubation to determine the concentration needed to observe 50% induction of apoptosis. The experiment was repeated three times. Representative images from one experiment are shown in Figure 3a, and the averages of three independent experiments ± standard deviation are shown in Figure 3b. Dragmacidin D was also tested in the traditional 2D MTT assay using the same serial dilutions with a 72 h incubation for treatment. Dragmacidin D failed to show any cytotoxicity in the 2D MTT assay at any of the concentrations tested (Figure 3b in dark green). The IC_50_ for cytotoxicity based on caspase cleavage in the spheroid assay was 8 ± 1 µM in MDA-MB-231 cells and 16 ± 0.6 µM in MDA-MB-468 cells at 24 h. Since no cytotoxicity was seen in the 2D MTT assay at even the highest concentrations tested (40 µg/mL ≈ 75 µM), the IC_50_ for cytotoxicity under 2D conditions was found to be >75 µM (Figure 3c). 

While observing apoptosis within 24 h of treatment is not unusual, it did give us pause that only caspase 3 cleavage was observed to be induced even when using higher concentrations of the compound. Since there are marine natural compounds that exhibit autofluorescence, we first ruled this out by treating the spheroids with dragmacidin D but not using any of the stains of the assay prior to imaging. No autofluorescence was observed (Figure S1a). The assay was repeated with longer incubation times (48 h and 72 h), where the loss of cell membrane integrity and the loss of cell number began to be apparent in the images (Figure S1b) and in the quantitation (Figure S1c). These experiments confirmed that dragmacidin D was indeed inducing apoptosis in the TNBC spheroids.

**Figure 4:**
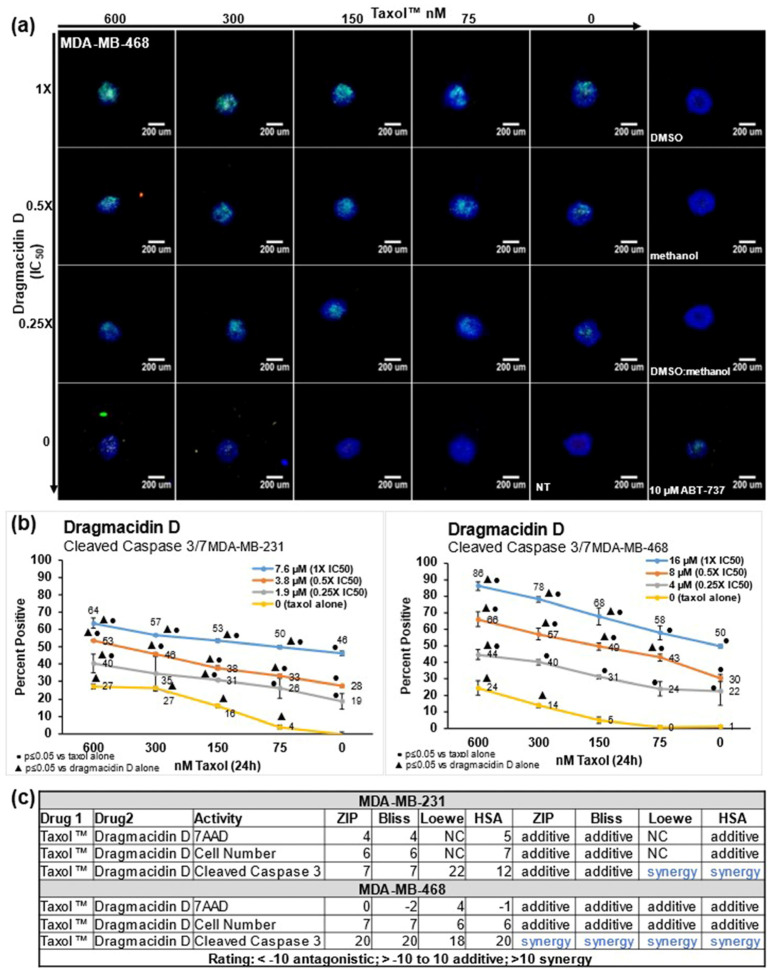
Dragmacidin D shows synergy with paclitaxel in the induction of apoptosis in TNBC cells. MDA-MB-231 and MDA-MB-468 spheroids were treated with 0, 75, 150, 300, or 600 nM paclitaxel and with 0, 0.25X, 0.5X, or 1X IC_50_ dragmacidin D for 24 h: (**a**) Images from one representative experiment for MDA-MB-468 cells are shown. Images were taken at 10× magnification. Scale bar is 200 µm. The increase in cleavage of caspase 3/7 produces green fluorescence, which is markedly increased in cells treated with the combination of treatments in a dose-dependent manner. (**b**) The average from three independent experiments ± standard deviation is shown in graph form. Statistical analysis was carried out using Student’s *t*-test with *p* ≤ 0.05. Paclitaxel treatment alone (yellow line) resulted in little caspase 3/7 cleavage. The combination of the treatments significantly enhanced the amount of caspase 3/7 cleavage observed in some of the combinations in the MDA-MB-231 and the MDA-MB-468 cells. (**c**) To determine if the enhanced response was additive or synergistic, the average of three independent experiments was analyzed in SynergyFinder 2.0. This program analyzes the data in 4 different models of synergy. The combination treatment with both paclitaxel and dragmacidin D resulted in synergy in the induction of caspase 3/7 cleavage in two of the four models of synergy used in the MDA-MB-231 cells, and in all four models of synergy for the MDA-MB-468 cells.

Because of the lack of targeted therapies against TNBC, chemotherapy is the first order of treatment. Paclitaxel is used as a treatment for triple-negative breast cancer, and, in some patients, it has a good response but overall exhibits a short progression-free survival (PFS) and low overall survival (OS) [18]. Nab-paclitaxel shows a better response, increasing the pathological complete response to 48.2%, which is almost twice that seen with paclitaxel (25.7%) [18]. Dragmacidin D was evaluated in combination with paclitaxel in the screening assay to determine if there was synergy with paclitaxel when treating the spheroids. Dragmacidin D was tested at 1, 0.5, 0.25, or 0X IC_50_ in each cell line. To those same wells, paclitaxel was added at 600, 300, 150, 75, or 0 nM. Images from one representative experiment for MDA-MB-468 are shown in Figure 4a. Images from one representative experiment for MDA-MB-231 are shown in Supplementary Figure S2a. The combination of dragmacidin D and paclitaxel exhibited more induction of apoptosis, as is clearly seen in the images with the increase in the green fluorescence that is caused by caspase 3/7 cleavage. The results of the average of three independent experiments for caspase 3/7 cleavage were graphed in Excel and are shown in Figure 4b for both cell lines. The graphs for the averages of three independent experiments for cell death measured by the intake of 7AAD and a decrease in cell number are shown in Supplementary Figure S2b for MDA-MB-231 cells and Supplementary Figure S2c for MDA-MB-468 cells. The caspase 3/7 cleavage graphs show that a much better response was achieved when the treatments were used in combination. To determine if this enhanced response was simply additive or synergistic, the average percentages of the three experiments were analyzed with free software called SynergyFinder 2.0 [19]. This software analyzes the data and provides a score using the Bliss, Zero interaction potency (ZIP), Loewe, and Highest single agent (HSA) models of synergy [19]. The HSA model quantifies the excess over the maximum single treatment response; the Bliss model looks at the multiplicative effect of treatments if they acted independently; the Loewe model looks at the expected response equivalent to an additive effect if the treatments were the same compound; and the ZIP model looks at the expected response corresponding to an effect if the treatments did not affect the potency of each other [19]. The data were analyzed in all four models of synergy, and the combination of paclitaxel and dragmacidin D exhibited synergy in the induction of caspase 3/7 cleavage in all these models of synergy in the MDA-MB-468 cell line (Figure 4c). An enhanced response was also seen in the MDA-MB-231 cell line, as shown in the graph (Figure 4b), but the results in this cell line were additive in the Bliss and ZIP models of synergy and showed synergy in the Loewe and HSA models (Figure 4c). 

**Figure 5:**
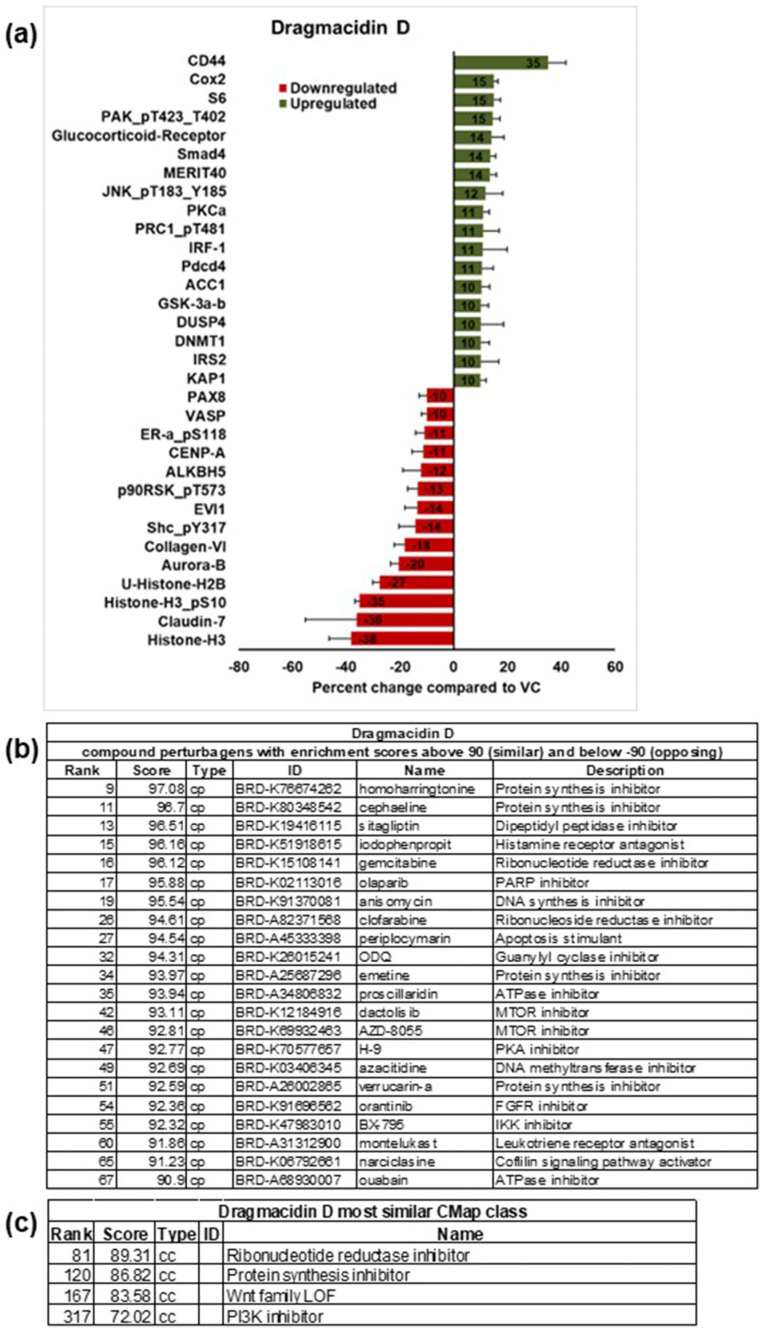
Effects of dragmacidin D in signal transduction and hypothesized modes of action. MDA-MB-231 cells with 2.5% matrigel were plated into all wells of a low adherence 384 well plate and spheroids were allowed to form overnight; then, half the plate was treated with 1× IC_50_ dragmacidin D or solvent control. After 24 h incubation, spheroids were harvested and pooled, and protein was extracted. The protein from three independent experiments was submitted to MD Anderson Reverse-Phase Protein Array Core Facility to be run in the same array. MDA Anderson ran the array and provided the resulting data: (**a**) The normalized data for the three independent experiments were averaged and compared to the control to provide the differential protein expression profile shown in the graph. Averages ± standard error of the mean are shown.. Only the averages of those proteins that changed more than 10% compared to solvent control are shown. (**b**) The list from this profile was entered into the Broad Institute Compare algorithm, and the resulting list of compounds most similar to dragmacidin D is shown for those whose score is ≥90% similar. (**c**) A list of the classes of compounds most similar to dragmacidin D is also provided, allowing for a hypothesis that dragmacidin D may be a ribonucleoside reductase inhibitor.

A reverse-phase protein array (RPPA) [20] was used to obtain insight into the changes in signaling induced by treatment with dragmacidin D. This array contains more than 450 antibodies representing important cell signaling pathways in cancer. Because dragmacidin D was more potent in the MDA-MB-231 cells, this cell line was chosen for this experiment. Spheroids were treated with 1X IC_50_ dragmacidin D or a methanol solvent control for 24 h, at which time the protein was extracted. The protein from three independent experiments was sent to the RPPA core at MD Anderson for testing and analysis. The core provided normalized data for the samples. The data from the three independent experiments were averaged, and the results for spheroids treated with dragmacidin D were compared to the solvent-control-treated spheroids to obtain a differential protein expression profile. The proteins that changed levels by more than 10% are shown in Figure 5a. The five most upregulated proteins were CD44 (35%), Cox2 (15%), ribosomal protein S6 (15%), p21-activated kinases (PAKs) phosphorylated at threonines 423 and 402 (15%), and the glucocorticoid receptor (14%). CD44 regulates many cellular functions, including cellular proliferation and metastasis [21]. Activated PAKs have been associated with cell cycle regulation and inflammation [22]. While both Cox2 and glucocorticoids are involved in the regulation of inflammation, Cox2 promotes it, while glucocorticoids suppress the production of inflammatory cytokines, including those regulated by Cox2 [23]. Ribosomal protein S6 regulates cell growth and proliferation [24]. Interestingly, many of the proteins most downregulated are histones. The five proteins most downregulated were histone H3 (38%), claudin 7 (36%), phosphorylated histone H3 at serine 10 (35%), ubiquitinated histone H2B (27%), and aurora kinase B (20%). Claudin 7 is an important component of tight junctions, a barrier that regulates the movement of solutes, ions, and water across and between cells [25]. Histone H3 phosphorylation by aurora kinase B is one of the hallmarks of mitosis [26]. The ubiquitination of histone H2B enhances nucleosome stability, which is of importance in the regulation of chromatin dynamics [27]. Histone depletion can lead to cell cycle arrest and changes in gene expression [28]. 

The list of proteins most upregulated and downregulated was entered into the Broad Institute’s Next Generation Connectivity Map, which contains the genetic profiles of over a million small molecules [29]. By comparing our data to those profiles, we matched them to compounds with similar profiles with known mechanisms of action. This allows one to postulate a hypothesis about the mechanism of action of small molecules. The compounds that best matched the profile of dragmacidin D are shown in Figure 5b. The top matches were protein synthesis inhibitors. Other matches of interest were ribonucleotide reductase inhibitors and mTOR inhibitors. A new feature of this analysis is that it now provides the class of the compounds that match. This is shown in Figure 5c. For dragmacidin D, the best match was ribonucleotide reductase inhibitors, although this was only an 89% match (the closer to 100%, the better the match). The list of proteins that changed more than 10% was also analyzed using the Search Tool in the Retrieval of Interacting Genes/Proteins (STRING) database [30]. A gene set enrichment analysis that identifies the proteins most significantly enriched through statistical methods was used. c-Jun N-terminal kinase family (JNK) was seen in many of the functional enrichments reported

**Figure 6:**
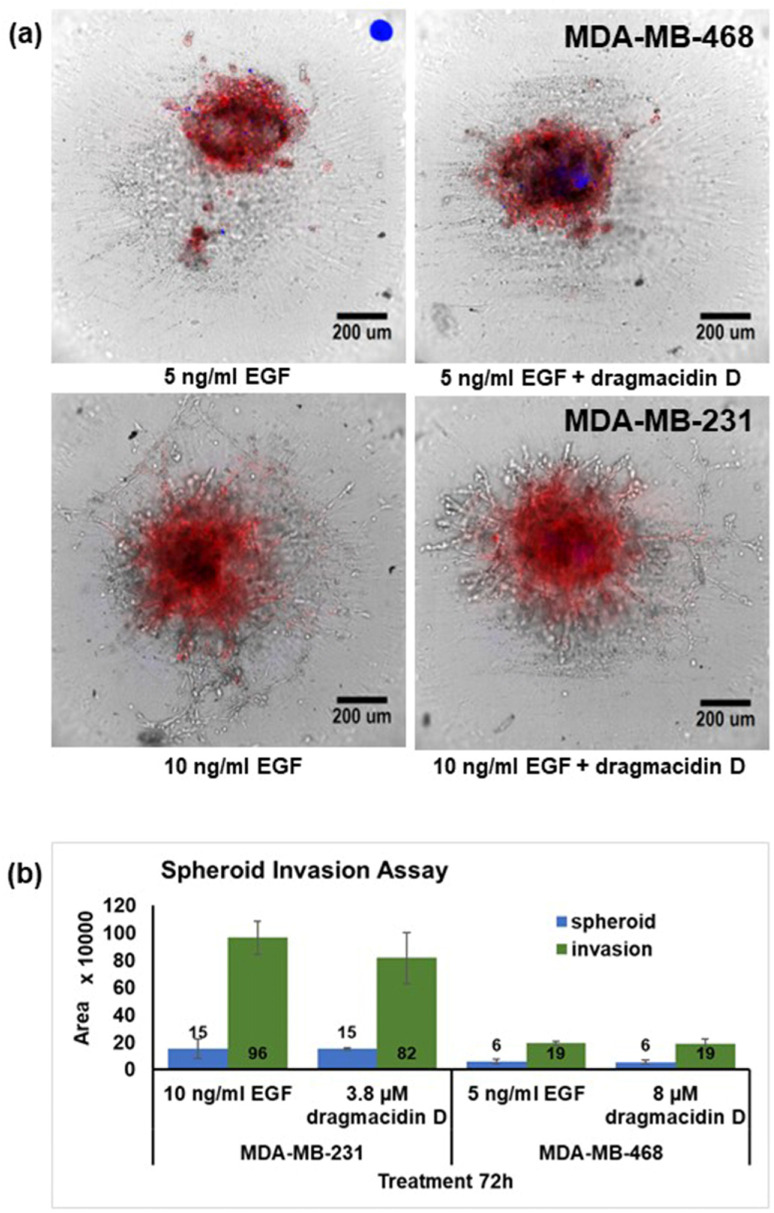
Effects of dragmacidin D in a spheroid invasion assay. Cells were plated and spheroids allowed to form overnight, with those from MDA-MB-231 using media containing only 5% FBS, while MDA-MB-468 cells received the normal 10% FBS media and then were encapsulated in matrigel supplemented with 5 or 10 ng/mL epidermal growth factor for the MDA-MB-468 and MDA-MB-231, respectively. The matrigel was allowed to set at 37 °C for 45 min when treatments of 0.5X IC_50_ dragmacidin D or media alone (complete media w/o phenol red at normal 10% FBS) were added. Spheroids cultured in the presence of EGF alone invaded the matrigel capsule: (**a**) Representative images of one experiment are shown. There was a slight decrease in the invasion when treatment of dragmacidin D was given. (**b**) The area of the spheroids and the invasion was measured, and the average of three independent experiments ± standard deviation is shown in the graph. While the perceived reduction in invasion was also captured in the measurements, the decrease failed to be statistically significant.

The upregulation of proteins that can foment cell growth and, in the case of CD44, metastasis led to the decision to assay for the effects of dragmacidin D in an invasion assay in vitro, as invasion is a marker for metastasis. A spheroid invasion assay was developed. Spheroids were encapsulated in matrigel containing the epidermal growth factor (EGF). After the matrigel set, treatments consisting of dragmacidin D at 0.5× IC_50_ for apoptosis in the spheroid assay in each cell line were added. This concentration was chosen because of the length of the treatment. The MDA-MB-468 cells migrated even in the absence of EGF, but the addition of 5 ng/mL EGF enhanced their migration. The pattern of migration for these cells was just an area of growth around the original spheroid. MDA-MB-231 cells required a higher concentration of EGF (10 ng/mL), as well as a gradient with 5% FBS-containing media, used when plating the spheroids, and 10% FBS-containing media, used when treating, to promote movement. The pattern of the migration of these cells is more traditional with lattice formation. Live cell imaging captured the images of the spheroids every 12 h, for a total of 72 h. Representative images from one experiment at 72 h are shown in Figure 6a. The area of the spheroids and the growth around them were scored, and the averages from three independent experiments are shown in Figure 6b. While dragmacidin D decreased the amount of invasion seen in the MDA-MB-231 spheroids, this decrease failed to be statistically significant using Student’s *t*-test. There was no discernible effect on MDA-MB-468 cells. Very importantly, no increase in invasion was observed despite the increase in CD44 expression.

## 3. Discussion

The marine natural product dragmacidin D shows antimicrobial, antiviral, and anti-inflammatory activity. As far as anticancer activity, to our knowledge, the only activity that was reported previously was cytotoxicity in the low-micromolar range at 72 h. While the activity reported here, 30 years later, is also in the micromolar range, there are three factors that set it apart. First, the activity is against TNBC cells grown as spheroids, which are known to require higher concentrations of drugs such as docetaxel, cisplatin, and epirubicin to observe an effect [15]. Second, the induction of apoptosis was observed with a short treatment time of 24 h, and third, while the induction of apoptosis was clearly observed in the spheroids at 24 h, no cytotoxicity was observed in the same cells when grown traditionally in 2D monolayer cultures even with a longer incubation (72 h) and at higher concentrations. Focusing on compounds that are active in 3D cultures with no activity in 2D allows for the detection of compounds whose activities would not have been found using traditional screening techniques and that are likely to act in a manner different from compounds found in those traditional assays.

The 3D culture of cancer cells is considered a more predictive model for the clinical assessment of the activity of compounds, as these spheroids better represent tumors, possessing nutrient and oxygen gradients, and having more interactions with the extracellular matrix and modified signaling. Spheroids also exhibit increased resistance to drugs and to apoptosis. Thus, the induction of apoptosis by dragmacidin D in spheroid cultures of both of the triple-negative breast cancer cell lines used is exciting. The reason why activity is observed against spheroids but not against 2D-grown cells is not known. It could be as simple as the fact that the compound is better transported into spheroids due to the changes in signaling caused by the cells growing as spheroids. The downregulation of claudin 7, which is an important factor in the tight junction regulation of the movement of solutes between and across cells, could suggest that this is a factor for dragmacidin D. Alternatively, it could be that if dragmacidin D has a molecular target, growing the cells in 3D upregulates the target or presents it in the right conformation for the compound to better interact with it. Furospinosulin 1, a compound we identified with activity in TNBC spheroids but not in 2D [13], requires hypoxic conditions for its activity to be observed [31]. Spheroids can also cause nutrient gradients, and some compounds show better activity under low-nutrient environments. Any of these is a possibility for dragmacidin D, and additional studies are warranted to understand why dragmacidin D has activity against spheroids but not against the same cells grown in 2D.

Paclitaxel is used as a treatment for triple-negative breast cancer, but patients tend to become resistant to it with repeated exposure [32]. Dragmacidin D showed synergy with paclitaxel in the induction of caspase 3/7 cleavage in both cell lines. This shows promise that dragmacidin D could be used in conjunction with paclitaxel to overcome resistance. Synergy was observed in both cell lines even when the lowest concentration of dragmacidin D (0.25X IC_50_) was used, but it was more pronounced in the MDA-MB-468 spheroids.

The use of a protein array that contains 450 different targets is a time-saving method for studying signal transduction changes at the level of protein over other techniques such as Western blotting. The changes in signaling caused by dragmacidin D are very different from those caused by furospinosulin 1, our previously identified compound [13]. The effects of dragmacidin D seem to involve fewer proteins, but the proteins appear to be more closely related, especially in the downregulation of histones or histone-regulating kinases. Not surprisingly, very different modes of action were hypothesized for each compound. The COMPARE algorithm suggests that the dragmacidin D signaling profile is similar to protein synthesis inhibitors, but it is difficult to understand why dragmacidin D would not have activity in 2D if this is the mode of action. The inhibition of protein synthesis could be the result of cell cycle arrest, an effect that would strongly correlate with the histone depletion observed. The other matches were ribonucleoside reductase inhibitors, mTOR inhibitors, and some anti-inflammatory compounds. Looking at the proteins present in the array for the PI3K/Akt/mTOR pathway, dragmacidin D appears to slightly activate this pathway rather than inhibit it (Figure S3). 

Of the twenty-two compounds with a Cmap score of 90 or greater, six have been reported to have activity in other cancer cell lines against the PI3K/Akt/mTOR pathway, five as protein synthesis inhibitors, three as protein kinase inhibitors, and three of the compounds as cell cycle inhibitors. In their description of the Connectivity Map (Cmap), the authors state that the hypothesized mechanism of action for small molecules is correct about 60% of the time [29]. It should also be taken into account that most of the profiles in the Cmap were obtained from cells grown in 2D culture. Thus, these modes of action may not be correct. 

The compounds derived from the COMPARE analysis shown in Figure 5b have a variety of biological activities and defined molecular targets, with 12 out of the 22 compounds being approved for clinical use in the US. Homoharringtonine (omacetaxine mepesuccinate, Synribo™) [33], anisomycin [34], narciclasine [35], and emetine [36] are inhibitors of protein synthesis. Homoharringtonine showed in vivo activity against aggressive, BRCA1/2 nonmutated TNBC cell lines with a rapid reduction in the antiapoptotic proteins Mcl-1, Bcl-2, survivin, and XIAP [37]. Anisomycin showed activity in mouse models of TNBC-inducing caspase-dependent apoptosis in TNBC cell lines. Anisomycin also targets angiogenesis and activates p38 and Jun N-terminal kinase (JNK) while decreasing mitochondrial membrane potential and adenosine triphosphate (ATP) levels, leading to the activation of 5′ adenosine monophosphate-activated protein kinase (AMPK) and the inhibition of the mammalian target of rapamycin (mTOR) signaling pathway [38]. Narciclasine inhibits TNBC cell proliferation and induces autophagy-dependent apoptosis in a dose-dependent manner. The apoptotic effects could be reversed using autophagy inhibitors, including an AMPK inhibitor and ULK1 siRNA. Narciclasine significantly inhibited TNBC tumor growth in mice by upregulating autophagy-dependent apoptosis [39]. It has also been shown to be a novel inhibitor of topoisomerase I [40]. Emetine has shown in vitro activity against the MDA-MB-231 and MDA-MB-468 TNBC cell lines [41]. It was shown to act via blocking Wnt/β-catenin signaling, resulting in a reduction in Wnt target genes. It also induced apoptosis and suppressed viability, migration, invasion, and sphere formation in TNBC cells [41]. 

The clinically used nucleosides gemcitabine and clofarabine (Clolar) are inhibitors of ribonucleotide reductase and DNA polymerase, resulting in the depletion of the levels of intracellular deoxynucleoside triphosphates available for DNA replication [42,43]. Homoharringtonine [44], periplocamine [45], dactolisib [46], AZD-8055 [47], anisomycin [38], verrrucarin A [48,49], and BX-795 [50] all have effects on the PI3K/Akt/mTOR pathway in different cancer models, contributing to their antitumor activities. 

Cephaeline, proscillaridin, and azacitidine (Vidaza^®^) all show epigenetic effects in cancer cells. Cephaeline is an inductor of histone H3 acetylation and an inhibitor of mucoepidermoid carcinoma (MEC) cancer stem cells [51]. Proscillardin A is a cardiac glycoside inhibitor of the Na(+)/K(+) ATPase (NKA) pump used clinically for heart failure. In a leukemia model, it led to the degradation of MYC and epigenetic reprogramming, including a significant loss of lysine acetylation in histone H3 and the downregulation of histone acetyltransferases [52]. The hypomethylating agent azacitidine showed activity in preclinical models of brain metastases of breast cancer. It increased apoptosis, inhibited Wnt signaling and angiogenesis, and decreased cell metastatic potential in a brain-colonizing cell line derived from MDA-MB-231 cells [53]. 

The compounds iodophenpropit, olaparib (Lynparza^®^), montelukast (Singulair^®^), and narciclasine show anti-inflammatory activity related to their anticancer activity. Iodophenpropit is a selective histamine H3 subtype antagonist [54], and the inhibition of the H3 receptor can lead to the activation of caspase 3/7 in breast cancer cells [55]. Olaparib inhibits poly(ADP-ribose) polymerase (PARP), thereby blocking the repair of single-strand DNA breaks in BRCA-1-containing cancers [56]. The inhibition of PARP by olaparib also leads to its anti-inflammatory activity [57]. Montelukast is a cysteinyl leukotriene receptor (CysLT) antagonist that has been reported to protect asthma patients from developing cancers, including breast and lung cancers, and has been shown to induce apoptosis in the MDA-MB-231 TNBC cell line by targeting CysLT [58]. Narciclasine, a potent protein synthesis inhibitor, has recently been shown to possess potent anti-inflammatory activity by blocking leukocyte–endothelial cell interactions and the downregulation of the TNF receptor 1 [59]. Orantinib (SU6668), a synthetic oxindole, is a potent inhibitor of vascular endothelial growth factor, fibroblast growth factor, and platelet-derived growth factor receptors, and has shown antiangiogenic effects and regression in human tumor xenografts [60]. Sitagliptin (Januvia^®^) is a dipeptidyl peptidase-IV (DPP-IV) inhibitor and has been shown to increase mitochondrial biogenesis in MDA-MB-231 TNBC cells, leading to a switch from Warburg metabolism to anti-Warburg effect. It induces apoptosis, reduces viability, decreases cell migration, and increases the sensitivity of the cells to treatment with doxorubicin [61]. 

Many of the most downregulated proteins are involved in cell cycle regulation. When cell cycle arrest occurs as part of cell fate regulation, it is not unusual for it to be followed by the induction of apoptosis. One of the major challenges of having compounds that are only active in 3D is that all validation assays need to be conducted in 3D. Attempts were made to ascertain cell cycle progression in spheroids treated with dragmacidin D during the course of the grant but failed. The dissociation of the spheroids to use 2D methods resulted in the loss of those cells most affected by the compound. Similarly, attempts to perform intracellular staining in spheroids to test the hypothesized mode of action were unsuccessful. Future research will be conducted to develop these models in 3D cultures. 

Analyzing the data through STRING seemed to show that the Jun kinase pathway was enriched. This is not surprising as the Jun kinase pathway is of importance in the regulation of apoptosis [62]. In a sense, this serves as a confirmation of the activity first identified for this compound in the spheroids.

Dragmacidin D is a member of a broad class of compounds called bisindole alkaloids, and a number of bisindoles have shown utility in the treatment of cancer. For example, the bisindole natural product staurosporine is a potent protein kinase inhibitor with broad-spectrum activity against cancer cell lines. It has been studied extensively based on its protein kinase C inhibitory activity [63], but it also shows multikinase activity. The staurosporine analog midostaurin, marketed as Rydapt™ and Tauitmo™, is approved for the treatment of newly diagnosed acute myeloid leukemia cases that have mutations in the FMS-like tyrosine kinase 3 (FLT-3) tyrosine kinase. Midostarin has been studied in triple-negative breast cancer cells, which lack FLT-3, and it has been identified as an inhibitor of aurora kinase B, leading to cell cycle arrest and apoptosis [64]. The vinca alkaloids vincristine and vinblastine are approved for the treatment of numerous cancers, including breast cancer. The primary mode of action of these chemotherapies is to bind tubulin and block microtubule formation, leading to cell cycle arrest [65]. Cruciferous vegetables such as broccoli, brussels sprouts, cabbage, and cauliflower contain bisindole 3,3′-diindolylmethane (DIM), which has shown anticancer effects in clinical studies [66], and it is used as a dietary supplement. Its mechanism of action is believed to involve NF-κB, Akt, Wnt, PI3K/Akt/mTOR, and AhR signaling [66]. DIM also shows anti-inflammatory [67] and antibiotic [68] activity and shares these activities with dragmacidin D. 

Dragmacidin D could serve as a tool molecule to better understand the role of some of the proteins that were differentially expressed from the RPPA array. Some of the proteins that increase may not be considered advantageous in a TNBC drug, while others may have dual roles. For example, CD44 showed the greatest upregulation in the array. CD44 is a stem cell marker for T cells and has different functions in different cells [69]. In breast cancer cells, CD44 has been characterized as both a stem cell marker associated with metastasis and poor prognosis and as a tumor suppressor based on its interaction with hyaluronan, which can promote apoptosis through the activation of caspase 3 and the inhibition of PI3k activation and Akt phosphorylation [70]. The results obtained in the invasion assay suggest that the increase in CD44 caused by dragmacidin D does not associate with an increase in invasion (a marker for metastasis), at least in vitro. This result, along with the induction of apoptosis, suggests that the increase in CD44 caused by dragmacidin D treatment is associated with antitumor effects. Further investigation is required to confirm this observation, but it opens intriguing opportunities for further use of dragmacidin D to study the duality of CD44 in breast cancer. 

## 4. Conclusions

Dragmacidin D, a known marine natural compound derived from a sponge, is shown herein to induce apoptosis in the triple-negative breast cancer cell lines MDA-MB-231 and MDA-MB-468 grown as spheroids (3D cell culture). However, it failed to induce cytotoxicity in the same cell lines with a longer incubation when they were grown in the traditional (2D) manner. Furthermore, dragmacidin D exhibited synergy with paclitaxel in the same screening assay. The changes in signaling observed show that dragmacidin D has a profound effect on histones, lowering their expression. Dragmacidin D also affected the molecules involved with inflammation and proliferation. The differential protein expression caused by treatment with dragmacidin D versus treatment with the solvent control in spheroids was used to hypothesize a mechanism of action, with protein synthesis inhibition, ribonucleotide reductase inhibition, PI3K inhibition, or Wnt family loss of function considered the possibilities. Further work is needed to determine if any of these hypotheses are correct, and why the compound only exhibits activity against cells grown in 3D.

While the selective induction of apoptosis in spheroids and the synergy with paclitaxel are sufficient to be enthusiastic about the compound, the decrease observed in in vitro invasion in the MDA-MB-231 spheroids versus the control is also interesting, although it failed to be statistically significant at the concentration tested. Nevertheless, future experiments could explore if the compound has effects on metastasis at other concentrations or through changes in cytokine and/or matrix metalloproteinase production.

Triple-negative breast cancers have limited therapeutic options. Thus, we believe the activities of dragmacidin D on TNBC spheroids reported here are promising and make this compound worthy of further research to define its therapeutic potential.

## 5. Materials and Methods

### 5.1. Dragmacidin D

The compound used in this study was obtained from the HBOI Pure compound collection. The sponge source, isolation protocol, and structure elucidation have been reported previously [1]. It was isolated as its trifluoroacetate salt. The stock material was analyzed using ^1^H and ^13^C NMR and high-resolution mass spectrometry prior to use in the studies reported here, and it was found that the material was >98% pure (Supporting Figures S4–S6). 

### 5.2. Reagents

The methanol used in the experiments was purchased from Fisher Scientific (Fair Lawn, NJ, USA). The 3-[4,5-Dimethyl-2-thiazolyl]-2,5-diphenyl-2H-tetrazolium bromide (MTT) used for cell viability assays, as well as 5-fluorouracil and 7 amino actinomycin D used in the screening assay, were purchased from Sigma Chemical Co. (St. Louis, MO, USA). Matrigel was purchased from BD Biosciences (San Jose, CA, USA). ABT-737 was purchased from Apexbio Technology, LLC. (Houston, TX, USA). Doxorubicin and paclitaxel were purchased from Calbiochem (now EMD Chemicals; San Diego, CA, USA). The human epidermal growth factor used in the invasion assay was purchased from Peprotech (Cranbury, NJ, USA).

### 5.3. Cell Culture

The human triple-negative breast cancer cell lines MDA-MB-231 (HTB-26) and MDA-MB-468 (HTB-132) were obtained from ATCC (Manassas, VA, USA), grown, aliquoted, and maintained in liquid nitrogen. Aliquots were thawed and grown in DMEM (including 4 mM L-glutamine, 4.5 g/L glucose, and 1.5 g/L sodium bicarbonate; ATCC 30-2002), supplemented with 10% fetal bovine serum (FBS; Hyclone SH3071, GE Healthcare/life sciences, Logan, UT, USA), 100 U/mL penicillin, G sodium, and 100 μg/mL streptomycin sulfate (Gibco, Carlsbad, CA, USA). Cells were maintained in a humidified incubator at 37 °C and 5% CO_2_. Cells were kept in culture for 10 weeks (20 passages) when a new aliquot was thawed.

### 5.4. The 3D Spheroid Multiparametric Assay

The conditions for spheroid formation for these cell lines were previously published [71] and were followed for this assay. The assay is based on a previously published assay [72], with modifications in terms of the stains used, incubation times, and the cells used. The assay in its current form was previously described [13], with changes in incubation times and solvents used. Briefly, MDA-MB-231 or MDA-MB-468 cells were plated on a black low-adherence spheroid 384-well tissue culture plate with a clear bottom (Corning 3830; Corning, NY, USA) at a concentration of 1500 cells per well in ice-cold complete media without phenol red containing 2.5% matrigel (BD Biosciences, Billerica, MA, USA) in a final volume of 30 μL/well and allowed to form spheroids overnight. After confirming the formation of a spheroid, 30 μL of a medium containing treatment at two times the final concentration was added. Dragmacidin D was tested at 5 μg/mL. Plate controls included 10μM ABT737, 5 μg/mL 5-fluorouracil, 0.5 μg/mL doxorubicin, media alone, and solvent controls. Cells were incubated with treatment for 24 h. At the end of this incubation, 20 μL/well of a staining mixture containing 2 drops/mL NucBlue Live Cell Stain Hoechst 33342 (Molecular Probes, Eugene, OR, USA), 5 μM CellEvent™ Caspase 3/7 Green Detection Reagent (Molecular Probes, Eugene, OR), and 100 μg 7-amino-actinomycin D (7AAD; Sigma, St. Louis, MO, USA) were added and allowed to incubate for 3 h. Cells were fixed with 4% paraformaldehyde for 30 min at 37 °C. Images were acquired using the ImageXpress^®^ Micro XLS widefield HCS (Molecular Devices, Sunnyvale, CA, USA), with a 10× Plan Fluor objective, binning at 2 and focusing on plate bottom and then offset by bottom thickness. A stack of 8 images separated by 10 μm was acquired, starting at the well bottom and covering approximately the lower half of each spheroid. The best focus projection of this stack was analyzed using the Multi-Wavelength Cell Scoring Module of the MetaXpress 5.1.0.3 software (Molecular Devices, Sunnyvale, CA, USA). The results were plotted using Microsoft Excel (Redmond, WA, USA). To normalize the results for 7AAD and caspase 3/7 cleavage, the solvent control values were subtracted from values for treatment samples. Total cell count was expressed as a percentage, comparing treatments to respective solvent controls. Compounds were tested in duplicate within plates, and hits were confirmed by repeating the testing.

### 5.5. The 2D Cell Viability Assay (MTT)

Cells were plated on a clear flat-bottomed 384-well tissue culture plate at a concentration of 3000 cells per well in a volume of 30 μL/well and allowed to adhere overnight. At the end of this incubation, 30 μL of medium containing treatment at two times the final concentration was added. Dragmacidin D was tested at 5 μg/mL. Other samples tested included 100 ng/mL super killer TRAIL and the known inducer of apoptosis microsclerodermin A at a concentration of 2.4 μg/mL [73]. Plate controls included 10μM ABT737, 5 μg/mL 5-fluorouracil, 0.5 μg/mL doxorubicin, media alone, and solvent controls. The same compounds were tested in the 2D assay at the same concentrations used in the 3D assay. The cells were then incubated for 72 h at 37 °C and 5% CO_2_. After this incubation, 125 μg of MTT was added to each well. The cells were then incubated for 3 h at 37 °C followed by centrifugation. The supernatant was removed, and 100 μL of acidified isopropyl alcohol (1:500 solution of hydrochloric acid to isopropanol) was added to each well to dissolve the crystals. The absorbencies of these solutions were measured at 570 nm with a plate reader (NOVOstar, BMG Labtech Inc., Durham, NC, USA). The resulting absorbencies were normalized against ethanol-treated cells using Microsoft Excel (Redmond, WA, USA). 

### 5.6. IC_50_ Determination

The normalized percentages from the spheroid assay were used to determine the dose needed to induce 50% cell death in the spheroid assay or the 2D viability assay (IC_50_), using a nonlinear regression curve fit with GraphPad Prism 5 software (La Jolla, CA, USA). For dragmacidin D spheroids, the values reported are based on the induction of caspase 3/7 cleavage. The reported values represent the average from three independent experiments ± standard deviation.

### 5.7. Combination Experiments with Paclitaxel and Synergy Determination

The above-described spheroid assay was used to determine if the combination of the compound with paclitaxel would induce synergy. Dragmacidin D was tested at 1, 0.5, 0.25, or 0X IC50 in each cell line. To those same wells, paclitaxel was added at 600, 300, 150, 75, or 0 nM. Cells were incubated with treatments for 24 h. The averaged percentages from three independent experiments in the screening assay were entered into the web application SynergyFinder 3.0 [19], which analyzes drug combination data in the Zip, Bliss, Loewe, and HAS models of synergy. It provides a numerical rating for each of these models. If the score has a value less than −10, the interaction is deemed antagonistic. If the score is greater than −10 but less than 10, the interactions are deemed additive. A score greater than 10 denotes a synergistic interaction. For this publication, the data were analyzed in all models. NC denotes that a score was not calculated in a particular model.

### 5.8. The 3D Spheroid Invasion Assay

The invasion assay conditions used in this assay were adapted from a previously published method [74] to work on a 384-well plate and with our cell lines. The EGF concentrations used in this assay were based on a previous publication [75]. MDA-MB-231 or MDA-MB-468 cells were plated on a black low-adherence spheroid 384-well tissue culture plate with a clear bottom (Corning 3830; Corning NY) at a concentration of 1500 cells per well in ice-cold complete media without phenol red (MDA-MB-468) or 5% FBS complete media without phenol red (MDA-MB-231), containing 2.5% matrigel (BD Biosciences, Billerica, MA, USA) in a final volume of 30 μL/well and allowed to form spheroids overnight. After confirming spheroid formation and that spheroids were centered, 15 μL/well of cell stain mix was added, and spheroids were incubated for 3 h at 37 °C. The cell stain mixture consisted of 2 drops of NucBlue Live Cell Stain Ready Probes (Molecular Probes R37605, Eugene, OR, USA) and 1µL of CellMask™ Deep Red Actin Tracking Stain (Invitrogen A57248, Carlsbad, CA, USA) stock in 500 µL of complete media without phenol red. At the end of the incubation, the plate was placed on the hood to cool down. Spheroids were encapsulated in 25 μL/well matrigel containing the epidermal growth factor, using 5 ng/mL for MDA-MB-468 and 10 ng/mL for MDA-MB-231. NT spheroids were just encapsulated in matrigel to serve as a negative control. Cells were incubated at 37 °C for 45 min. Treatments consisting of 0.5× IC_50_ in the spheroid assay were added in 30 µL of complete media without phenol red. The plate was placed on the high-content imager environmental chamber at 37 °C with 5% CO_2_ for live imaging. A stack of images was taken for each wavelength at each time point, with the best focus projection of this stack being saved. Images were acquired every 12 h for 72 h (0, 12, 24, 36, 48, 60, and 72) using the ImageXpress^®^ Micro XLS widefield HCS (Molecular Devices, Sunnyvale, CA, USA), with a 20X Plan Fluor objective. The 72 h best-focus projection image for transmitted light was used for analysis using MetaXpress 5.1.0.3 software (Molecular Devices, Sunnyvale, CA, USA). The area around the spheroids and the invasion was drawn using the trace region tool. Region statistics were logged onto Microsoft Excel. The resulting areas from three independent experiments were averaged and graphed using Excel.

### 5.9. Reverse-Phase Protein Array (RPPA)

MDA-MB-231 cells were plated and allowed to form a spheroid overnight in a 384-well low-adherence spheroid plate. All wells had cells. Half the cells on the plate were treated with methanol (vehicle control) or 1X IC_50_ dragmacidin D for 24 h. At the end of treatment cells were harvested, pooled, and protein was isolated using RPPA lysis buffer (1% Triton X 100; 50 mM HEPES; pH 7.4; 150 mM NaCl; 1.5 mM MgCl_2_; 1 mM EGTA; 100 mM NaF; 10 mM Na pyrophosphate; 1 mM Na_3_VO_4_; 10% glycerol; and protease and phosphatase inhibitors from Roche Applied Science Cat. # 05056489001 and 04906837001, respectively). Protein was adjusted to a concentration of 1.5 µg/µL, and the protein from three independent experiments was submitted to MD Anderson Reverse-Phase Protein Array Core Facility so that samples were tested in the same array. Samples were treated according to their published methods [20,76]. Briefly, samples were serial-diluted and printed on nitrocellulose-coated slides. Slides were probed with 450 validated primary antibodies, followed by detection with appropriate biotinylated secondary antibodies. Signals were detected through 3, 3′-diaminobenzidine (DAB)-horse radish peroxidase (HRP) colorimetric reaction. Spot density was determined using Array-Pro Analyzer 6.3 software (MediaCybernetics, Rockville, MD, USA), and protein concentration was determined via super-curve fitting. As part of the service, MDA Anderson provided a data report that included raw, normalized, and median-centered data, as well as a heat map. The normalized data from the three independent experiments were averaged, the standard error of the mean was calculated, and differential protein expression obtained comparing the averages from dragmacidin D-treated spheroids to solvent-control-treated spheroids were graphed using Microsoft Excel. 

## Data Availability

Any data not included in the manuscript or supplementary materials that support the work presented in this manuscript are available upon reasonable request to the corresponding author (E.A.G.).

## Supplementary Materials

The following supporting information can be downloaded at: https://www.mdpi.com/1660-3397/21/12/642/s1, Figure S1: Lack of autofluorescence at 24 h and markers of late apoptosis seen with treatment of TNBC spheroids with dragmacidin D with 48 h and 72 h incubations; Figure S2: Synergy testing in MDA-MB-231 and graphs for decrease in cell number and loss of membrane integrity for MDA-MB-468; Figure S3. Differential protein expression of dragmacidin D-treated spheroids compared to solvent-control-treated spheroids for proteins of the PI3K/Akt/mTOR pathway represented in the RPPA array; Figure S4. 1H NMR spectrum of dragmacidin D; Figure S5. 13C NMR spectrum of dragmacidin D; Figure S6. Mass spectrum of dragmacidin D.
