# Supplementary material for: The Marine Natural Compound Dragmacidin D Selectively Induces Apoptosis in Triple-Negative Breast Cancer Spheroids
Source: Mar Drugs. 2023 Dec 15;21(12):642. doi: 10.3390/md21120642 (PMC10871089; doi:10.3390/md21120642)
Supplement: Supplementary file 1 [file marinedrugs-21-00642-s001.zip › marinedrugs-2746551-supplementary.pdf]

Figure S1. Lack of autofluorescence at 24h and markers of late apoptosis seen with treatment of TNBC spheroids with dragmacidin D with 48h and 72h incubations**.** **(a)** Representative images of one experiment in MDA-MB-231 cells treated with dragmacidin D for 24h without staining mix showing dragmacidin D has no autofluorescence and (**b)** Representative images of one experiment MDA-MB-468 cells treated with dragmacidin D for 48 or 72h with staining mix showing cells exhibiting markers of late apoptosis and cell death (loss of membrane integrity and decrease in cell number) with (**c)** graphs for both cell lines of 48h and 24h data. Pictures were taken at 10X magnification. Scale bar is 200 µm. Blue: nuclei (Hoechst 33342), Red: loss of membrane integrity (7-aminoactinomycin D), Green: cleaved caspase 3/7

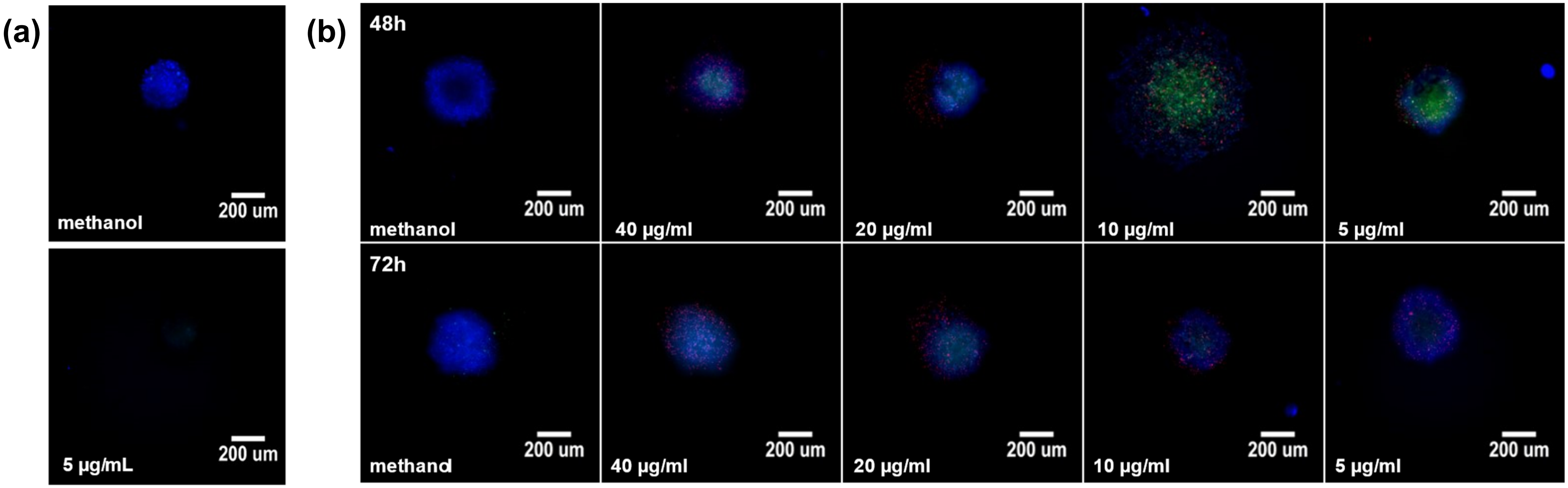

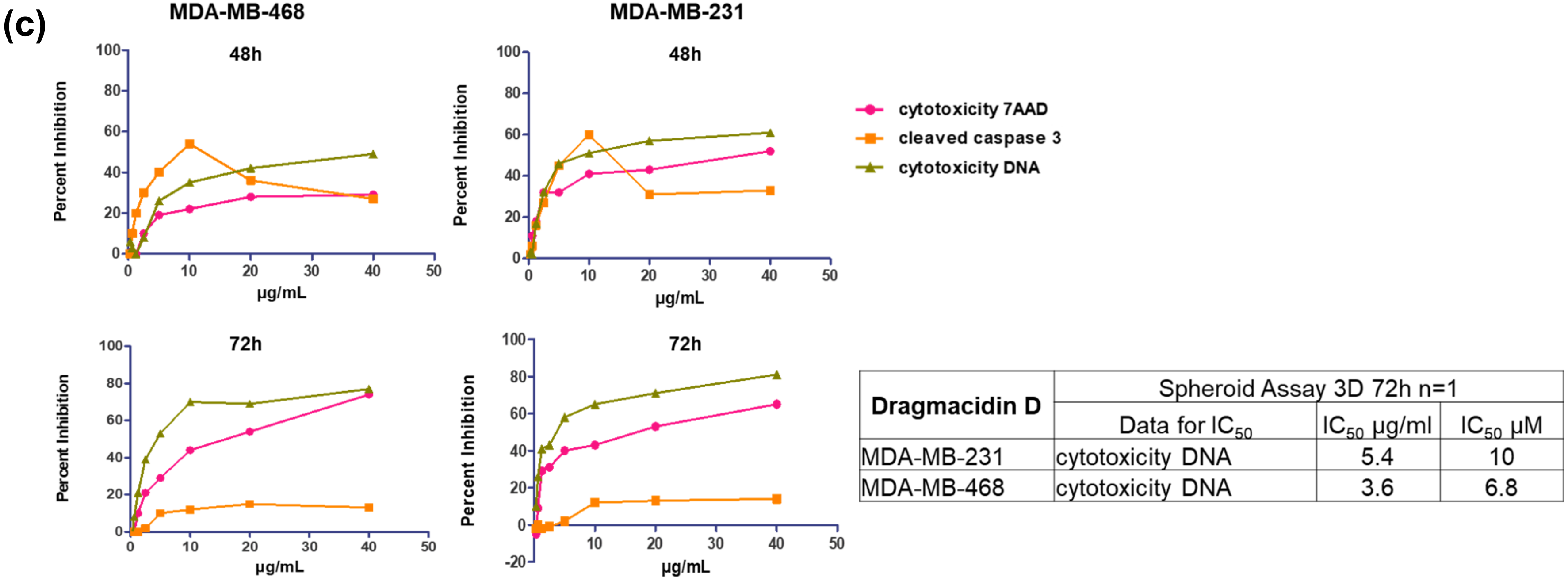

Dragmacidin D	Spheroid Assay 3D 72h n=1		
	Data for IC_{50}	IC_{50} µg/ml	IC_{50} µM
MDA-MB-231	cytotoxicity DNA	5.4	10
MDA-MB-468	cytotoxicity DNA	3.6	6.8

Figure S2. Synergy testing in MDA-MB-231 and graphs for decrease in cell number and loss of membrane integrity for MDA-MB-468. (**a**) Representative images of one synergy experiment in MDA-MB-231 cells, and average graphs of 3 experiments for decrease in cell number and 7AAD measurements in (**b**) MDA-MB-231 and (**c**) MDA-MB-468 cells. Pictures were taken at 10X magnification. Scale bar is 200 µm. Statistical analysis was performed using a Student's t-test where significance was determined with p ≤ 0.05.

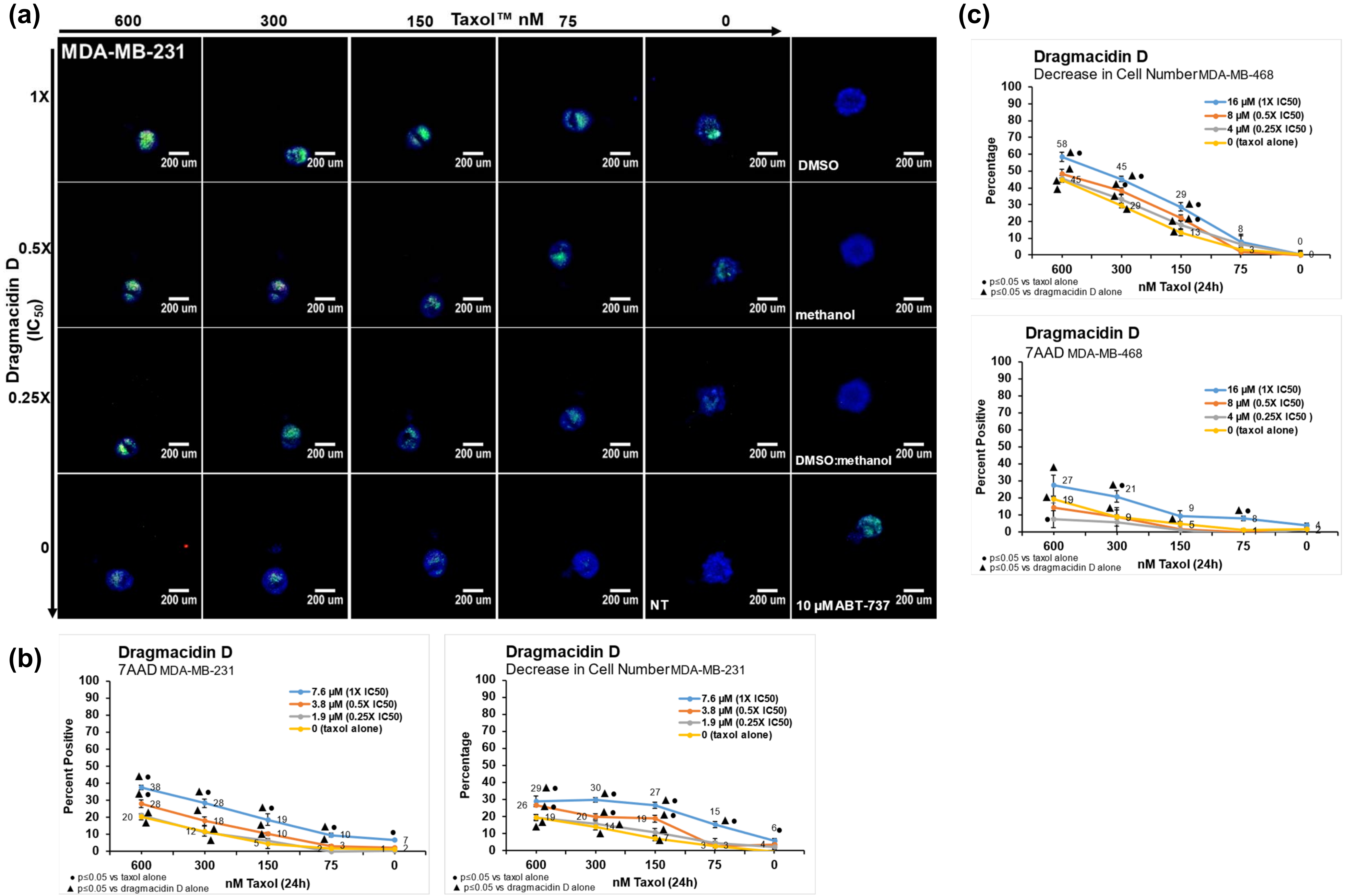

Figure S3. Differential protein expression of dragmacidin D treated spheroids compared to solvent control treated spheroids for proteins of the PI3K/Akt/mTOR pathway represented in the RPPA array. The graphs represent the average of 3 independent experiments ± standard error of the mean.

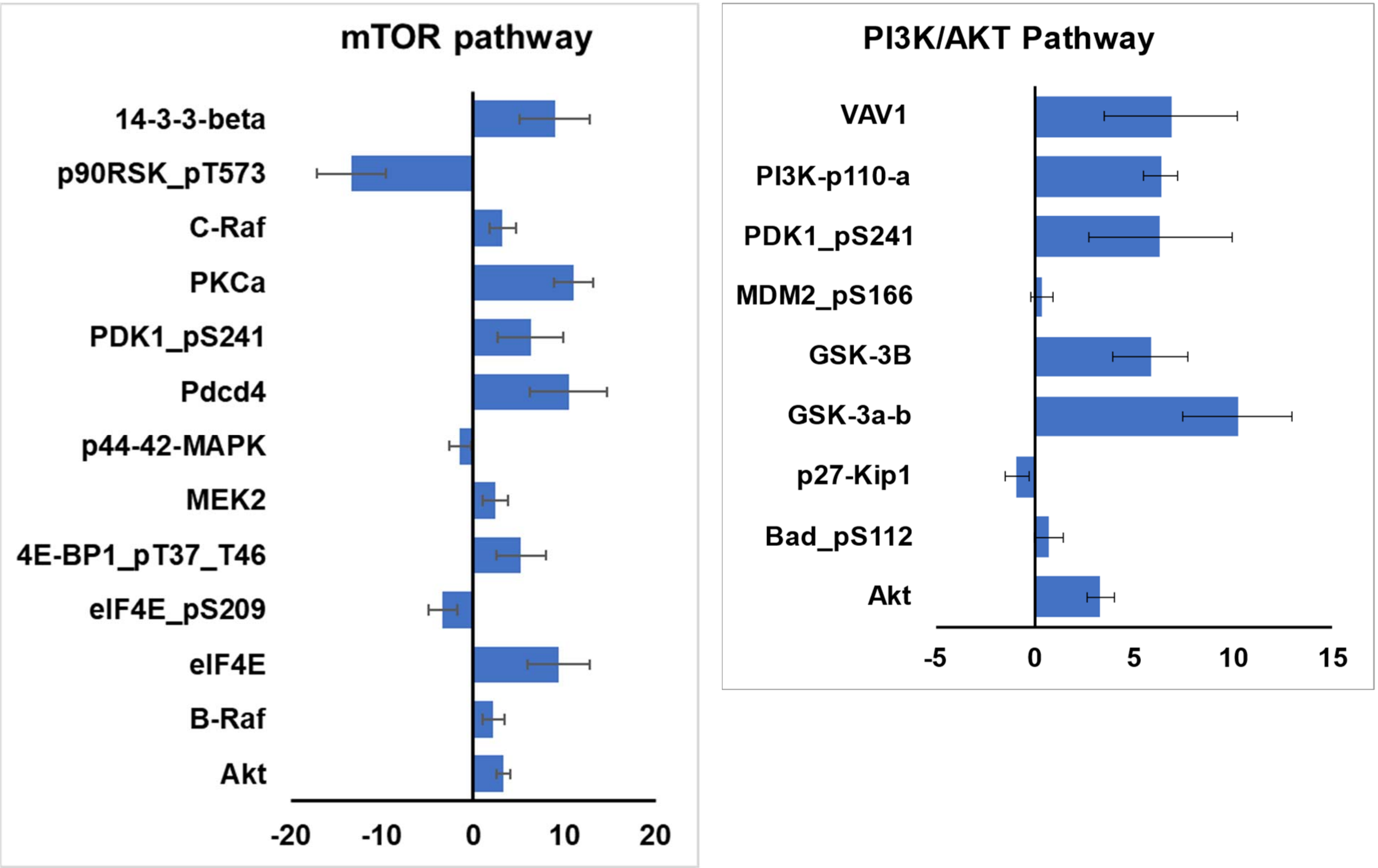

Figure S4. ^{1}H NMR spectrum of dragmacidin D used in the study. (d_4-methanol, 600 MHz)

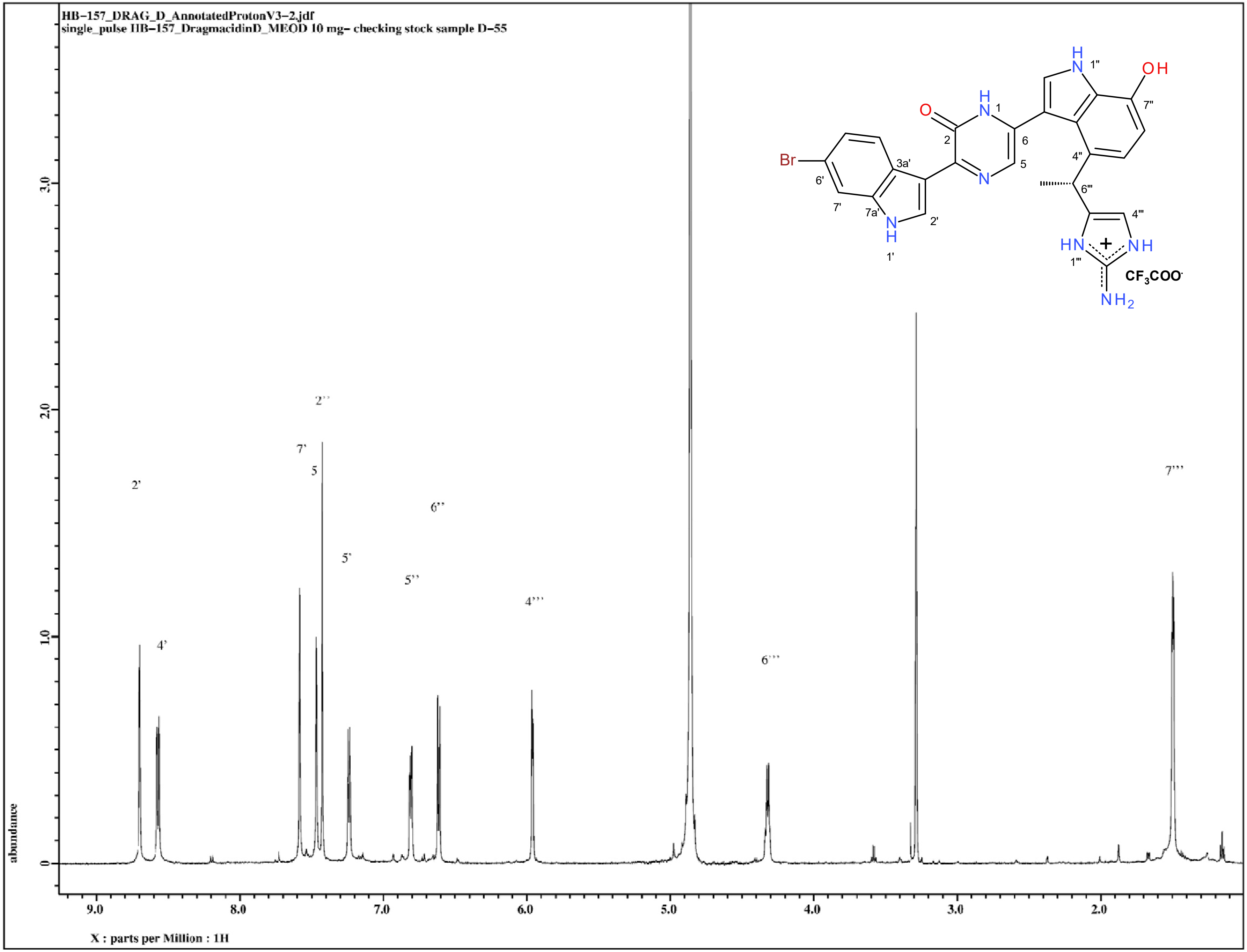

Figure S5. ^{13}C NMR spectrum of dragmacidin D used in the study. (d_4-methanol, 150 MHz)

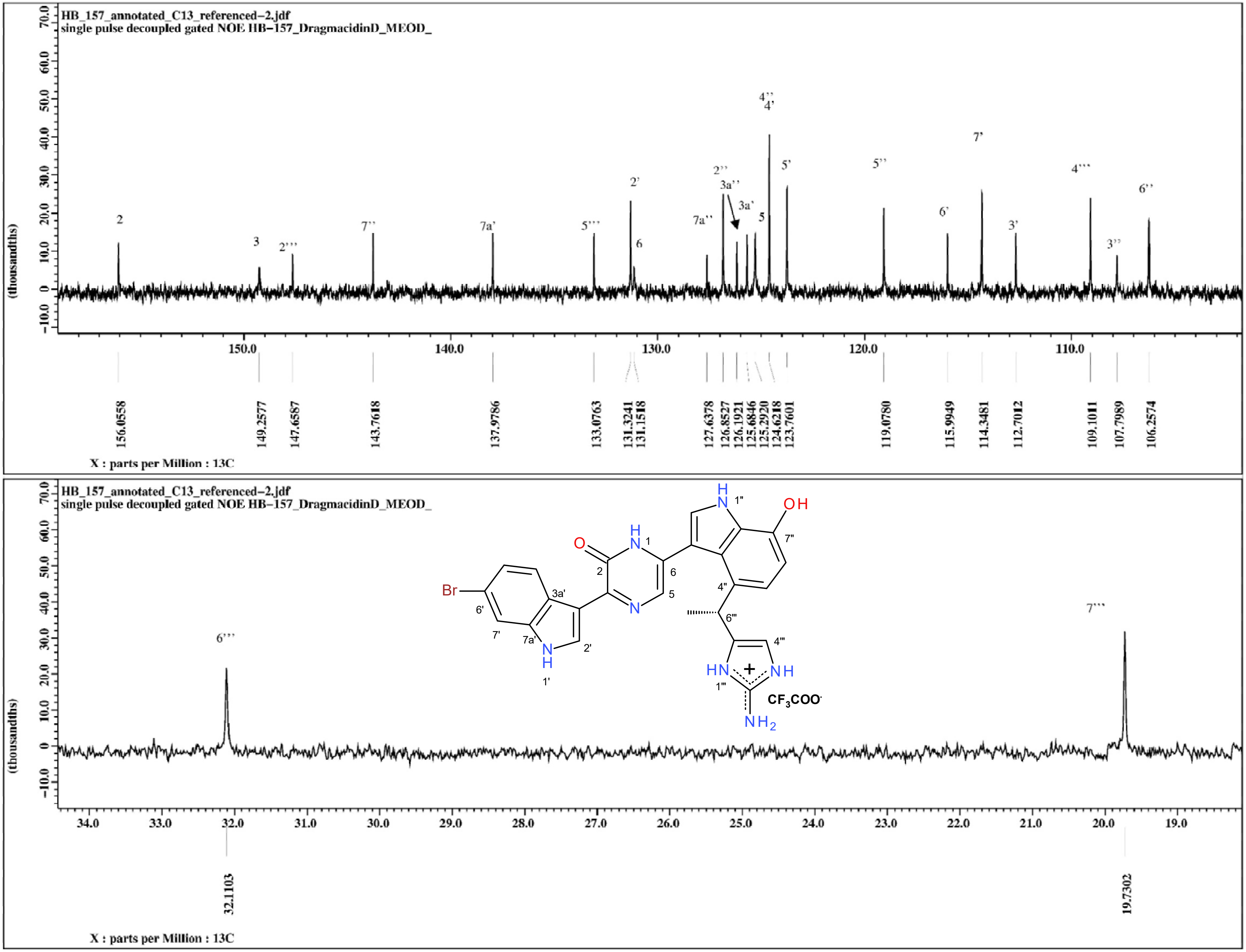

Figure S6. Direct infusion ESI + mode mass spectrum of dragmacidin d used in the study.

Elemental Compositions

D:\msAxel@LP Data\Amy data\Samples\HB-158_DragmacidinD_ESI+.txt

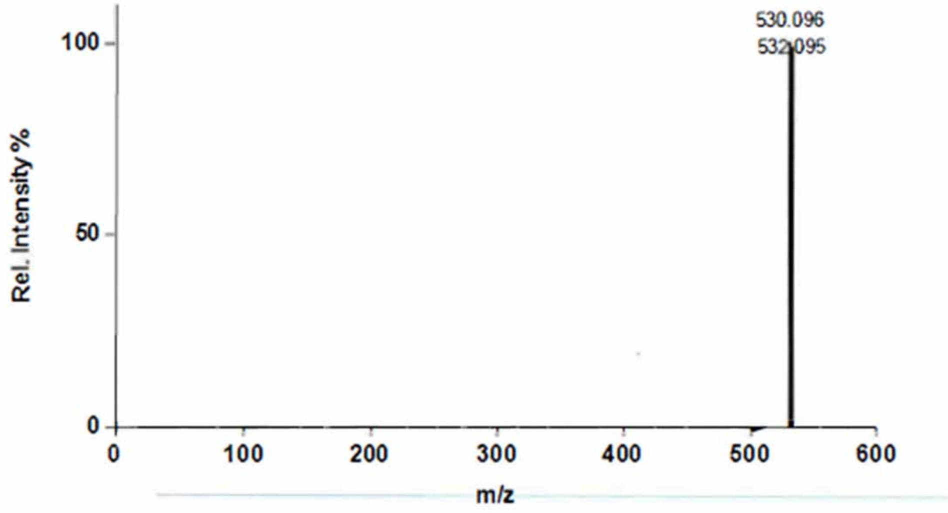

Mass Spectrum

Elemental Compositions
Element Limits: C 0/50 H 0/100 O 0/10 N 0/10 Br 1/1
Tolerance: 5 mmuEven or odd electron ion or both: Even
Electron correction: None.Charges: 1
Minimum unsaturation: -1Maximum unsaturation: 100

Calc. m/z	Abund %	mmu	DBE	Composition
530.095859	100.000	0.28	5.5	C13H25O9N9Br1
530.099882	100.000	4.30	9.5	C18H25O7N7Br1
530.092673	100.000	-2.91	13.5	C24H25O6N3Br1
530.094009	100.000	-1.57	18.5	C25H21O2N7Br1
530.096696	100.000	1.12	17.5	C29H25O4N1Br1
530.098032	100.000	2.45	22.5	C30H21N5Br1
532.090380	98.609	-4.65	9.5	C16H23O7N9Br1
532.093067	98.609	-1.97	8.5	C20H27O9N3Br1
532.094403	98.609	-0.63	13.5	C21H23O5N7Br1
532.097090	98.609	2.06	12.5	C25H27O7N1Br1
532.098426	98.609	3.39	17.5	C26H23O3N5Br1
532.091217	98.609	-3.82	21.5	C32H23O2N1Br1

The formula of DragmacidinD is C25H21BrN7O2
